# Supplementary material for: Applying the principles for digital development to improve maternal and child health in the Peri-urban areas of Karachi, Pakistan
Source: PLOS Digit Health. 2024 Jan 29;3(1):e0000434. doi: 10.1371/journal.pdig.0000434 (PMC10824452; doi:10.1371/journal.pdig.0000434)
Supplement: Attachment — Submitted filename: Digital Development_Response to Reviewers.docx [file pdig.0000434.s001.docx]

**Title of Manuscript: Applying the Principles for Digital Development to Improve Maternal and Child Health in the Peri-Urban Areas of Karachi, Pakistan [PDIG-D-23-00291]**

Dear Reviewers,

Thank you for your valuable comments on our manuscript. Below are our responses to the different points raised during the review process. All revisions are in track changes within the revised manuscript document.

Please note that the reference line and page numbers mentioned below are from the marked version of the revised manuscript, with tracked changes (“All Markup” view).

|  | **Reviewer Comment** | **Author Response** |
| --- | --- | --- |
|  | **Reviewer 1** | |
| **1** | This is an excellent descriptive study of the implementation of electronic tools to improve maternal and child health outcomes. The study and implementation were done based on prior analysis of existing tools and needs. The manuscript is informative, well-outlined, and clear.  The figures are almost unreadable, they need to be redone or resubmitted. Figure 4 appears to be the lifecycle of the work. It will benefit from arrows to indicate the progression and its own feedback. | We thank reviewer 1 for their helpful comments. All figures have now been re-drawn and downloaded in higher resolutions (300 dpi) to ensure better readability. |
|  | **Reviewer 2** | |
| **1** | Addressing MNCH in the LMI group of nations is important to bridge up the healthcare divide. Therefore, the study is appropriate and pertinent to the present LMI nations' healthcare delivery. Although the study is well-structured, there are the following opportunities to improve it further,  Incorporation of Qualitative and Quantitative analysis with reference to end users (demographic and SDoH parameters, comorbidities, addictions, practice of safe sex, etc.) | We thank reviewer 2 for their helpful comments. Understanding the user context is very important as we implement DHIs.  To clarify, our end-users are frontline health workers (e.g., community health workers, midwives, allied health workers, vaccinators, physicians), not patients. The parameters that have been mentioned are more relevant for patient-facing applications. However, we have highlighted the demographic profile of our user base in the revised manuscript –**see lines 140 to 145 on page number 6 of the manuscript.** |
| **2** | A separate section on how the proposed DHI method has helped generate awareness in the society and how it is measured | We agree that it is important to share the DHI’s impact on the community –a section labelled ‘DHI Impact’ has been added where we share how the findings from the DHI have informed the operations of health workers and supplemented the impact of their activities, **see lines 208 to 221 on pages 9-10.** |
| **3** | Authors may be benefited by reading the following articles, which they may cite in this work to strengthen its foundation:  Chattopadhyay S., Daneshgar F. – “An Awareness Net Collaborative Model for Schizophrenia Management”, International Journal of Advanced Intelligence Paradigms (2013); 5(3): 217-232.  Acharya U. R., Faust O., Ghista D. N., Sree V. S., Alvin A.P.C., Chattopadhyay S., Lim T-C., Ng E. Y. K., Yu W.Y – “A Systems Approach to Cardiac Health Diagnosis", International Journal of Medical Imaging and Health Informatics (2013); 3: 1-7.  Chattopadhyay S., – “A Prototype Depression Screening Tool for Rural Healthcare: A Step towards e-Health Informatics”, Journal of Medical Imaging and Health Informatics (2012); 2(3): 244-249  Chattopadhyay S., Saurabh R., Land L., Acharya U. R. – “Studying Infant Mortality Rate: A Data Mining Approach”, Health and Technology (2011); 1(1): 25-34  Daneshgar F., Chattopadhyay S. – “A Framework for Crisis Management in Developing Countries”. Intelligent Decision Technologies: an international journal (2011); 5(2): pp. 189-199.  Chattopadhyay S. –"A Framework for Studying Perceptions of Rural Healthcare Staff and Basic ICT Support for e-health Use: An Indian Experience". Telemedicine and e-Health (2010); 16(1): pp. 80-88.  Li J, Land L.P.W, Ray P. Chattopadhyay S. –"E-Health Readiness Framework from Electronic Health Records Perspective". International Journal of Internet and Enterprise Management: Special Issue in Healthcare (2010); 6(4): pp. 326-348  Ray P., Chattopadhyay S. – “Fuzzy Awareness Model for Disaster Situations”. Intelligent Decision Technologies: an international journal [Special Issue on Intelligent Decision Making in Dynamic Environments: Methods, Architectures and Applications] (2009); 3 (1): pp. 75-82. | Thank you for sharing this insightful literature. We have cited the relevant literature on **lines 370–379 on page 17.** |
|  | **Reviewer 3** | |
| **1** | Thank you kindly for the opportunity to read through the paper entitled, “Applying the principles for digital development to improve maternal and child health in the peri-urban areas of Karachi Pakistan.” This paper is well-written and easy to follow and describes important work in using novel digital technologies to improve the health of women and children. I have made a few comments regarding how this paper might be strengthened below.  First, I would suggest that the Principles for Digital Development section in the Materials and Methods be moved to the Introduction section. I think an upfront discussion of these principles and why they are appropriate to guide the development of the DHI presented in this paper is important. | We thank reviewer 3 for their helpful comments.  We have moved the section to the introduction as per this comment. We have also elaborated on how the principles can be applied to different fields within development.  **See lines 93 to 103 on page 4.** |
| **2** | I would also recommend adding more details to describe the tool. In particular, I think it should be abundantly clear when reading through the Materials and Methods what comprises this tool and how its functionalities might improve maternal and Child health. | Thank you for pointing this out. We’ve revised the description of DHI and elaborated on both the functionalities, user roles, and flexibility of the platform to meet project/research requirements.  **We have elaborated on the system’s functionalities and their impact in lines 175-186, page 8.** |
|  | a) For instance, I looked to Figure 1 and noted a discussion of a triage component of healthcare providers as well as a care coordination component, but it was not clear to me how these pieces of healthcare workflow are supported (if at all) by this DHI. | **For point 2a, see lines 146 to 160 on pages 6-7.** |
|  | b) I was also not clear as to what the 14 integrated modules involve. More detail might also be added regarding how this DHI can be customized to project requirements and objectives. Can detail be provided on which projects the team might be describing here? | **We have added more details on what the system modules involve, see lines 136-140, page 6; lines 153-160, page 7.**  **We have also added more details regarding how this DHI can be customized to project requirements. Please see lines 161-170, page 7.** |
| **3** | Throughout the Results section, I felt much more detail could be added. | Thank you for these valuable insights. We have addressed each comment in the manuscript. |
|  | a) For instance, in Section One Design with User, I was not clear as to how user-centered design was conducted in this particular instance. Who was involved in the process? How many people were involved in the process? When did the process occur? Where did the process occur? And how were users' opinions solicited? | Thank you for pointing this out.  We have highlighted that the program and development teams engaged with the users through user observation and face-to-face interviews over a period of several months (**lines 227-232, page 10; lines 241-243, page 11).**  We have elaborated on how the DHI is managed, i.e., we employ an iterative approach that is consistently reliant on both project requirements and user feedback to improve the DHI’s functioning. We’ve also cited a user experience study we published in 2022 where we formalize findings from user feedback.  **See lines 253 to 262 on pages 11—12.** |
|  | b) I also felt that this section did not highlight to any great degree how codesign principles were embedded in this project. I know some early consultations with users occurred, but how were users [if at all] continually engaged in the process over time? | We agree that this is essential information for our paper. We have touched upon this question in the **lines 246-251 and 253-262 on pages 11-12.** Our description of the user experience study and support process depicts how users have been engaged over time. |
|  | c) The sentence “the team has since been actively engaged in streamlining technological support to improve performance and experience with the new system” is vague to me. Details related to how this project was carried out are needed to improve the chances that it can support others aiming to engage in similar work. | We have elaborated on our user support strategy by describing how both tech and product support is available to end-users during field hours. See **lines 253 to 262 on pages 11-12.** |
|  | d) As another example, In Section Two Understand the Existing Ecosystem, the authors describe some findings related to local challenges, but it is not clear to me how these challenges were identified and prioritized by eventual DHI end users. Similar to the above, I am curious to know how these challenges were identified? Who was involved in the process? Etc. | Thank you for this valuable comment. We have explained how local challenges were identified in **lines 265 to 273 on page 12.** Challenges were identified and addressed through the technical expertise of our partners at AKU; AKU has been running a primary health center in the community since 2003 which has allowed them to gain deep experiential knowledge about the realities of field work and implementing healthcare services in low-resource settings. |
| **4** | Do the authors have any comments they can make on how well these processes worked? As the study was focused on implementation, I would like more details on who is using this product, how well it is being used, how care outcomes are changed, etc. | We agree that these are essential points to include in the manuscript. Reviewer 1 and 2 have also raised these questions –they have been addressed under the lines:  Who is using the DHI:  **Lines 138 to 145 on page 6**  How well it is being used/how care outcomes are change:  **Lines 207 to 221 on pages 9-10** |
| **5** | Overall, I think the authors have undertaken important work here. To maximize the potential impact this work can have on others interested in learning about or following the path of this team, many more details about the DHI in question, how this work was done, and the impact that it has had are needed. | Thank you for these insights. Reviewer 1 and Reviewer 2 also had similar points. We have elaborated more on the DHI, including the contextual need, details about the tool itself, development and user feedback process, and the impact it has created on the community.  See the following lines:  Contextual need: **118—124 on page 5**  Details about the tool: **136 to 170 on pages 6-7; 175 to 186 on page 8**  Impact: **207-221 on pages 9-10**  Development and user feedback process: **227-228, page 10; 232, page 10; 241-243, page 11; 253-262 on pages 11-12** |
