# Supplementary material for: Applying the principles for digital development to improve maternal and child health in the Peri-urban areas of Karachi, Pakistan
Source: PLOS Digit Health. 2024 Jan 29;3(1):e0000434. doi: 10.1371/journal.pdig.0000434 (PMC10824452; doi:10.1371/journal.pdig.0000434)
Supplement: Attachment — Submitted filename: Response to Reviewers_PDIG-D-23-00291R1.docx [file pdig.0000434.s002.docx]

**Title of Manuscript: Applying the Principles for Digital Development to Improve Maternal and Child Health in the Peri-urban Areas of Karachi, Pakistan [PDIG-D-23-00291R1]**

Dear Reviewers, Thank you for your valuable comments on our manuscript. Below are our responses to the different points raised during the review process. All revisions are in track changes within the revised manuscript document.

Please note that the reference line and page numbers mentioned below are from the marked version of the revised manuscript, with tracked changes (“All Markup” view).

|  | **Comment** | **Author Response** | |
| --- | --- | --- | --- |
|  | **Editor** | | |
| **1** | Although the topic of the manuscript is interesting, there are some major issues that need to be addressed: As noted by the authors “a narrative review of the design, development, and deployment of the DHI” has been presented in this manuscript. Therefore, the methods and results sections do not show any details for a research study and these headings should not be included in the manuscript.  Overall, it is better to present it as a case study and address all methodological principles for it. | | Thank you for highlighting this. We have presented the article as a case study and elaborated on its potential contribution to the evidence base for digital health for MNCH (see lines **110-117**, page **6**). The data used to formalize our learnings includes practice-generated observations and documentation, as highlighted in line **119**, page **6**.  Additionally, we have restructured the article as a case study, with the following section headings:   - Abstract (lines **20-44**, page **2**) - Author Summary (lines **45-59**, page **3**) - Introduction (lines **63-119**, pages **4-6**) - Presentation of the Case   - Implementation Context (lines **121-139**, page **7**)   - DHI Description (lines **140-209**, pages **8-11**)   - DHI Impact (lines **213-227**, page **12**)   - Intervention Elements (lines **228-361**, pages **12-18**) - Case Discussion (lines **362-444**, pages **18-22**) - Conclusion (lines **445-454**, page **22**) - Acknowledgements - References |
|  | **Reviewer 1** | | |
| **1** | The figures improved but the caption numbers on the figures are lost! Please put more thought into the figures. There is a lot of white space wasted and some of the figures provide very little information. Figure 2 might benefit from changing the layout to portrait with two screenshot views on each row and arrows to indicate the flow.  Remember the figures have to stand on their own and be informative. | Thank you for your feedback. We believe Reviewer 1 was referring to Figure 3 in the previous submission as that is the diagram that provided screenshot views. We have revised this figure (now **Figure 2: DHI features and screenshots)** by changing the layout to portrait mode. To clarify, this figure is meant to depict screens and features of the application (i.e., there is no flow). For improved clarity, we have added a figure legend to specify the feature each screenshot depicts.  After careful consideration, we have decided to supplement this figure with a detailed table outlining key features of the application (see lines **212-213**, page **11**).  We have also converted Figure 1 into a table, which better presents the 9 Principles (lines **92-93**, page **5**). **Figure 4** has been removed as it did not add much information and its content has been covered in the text in lines **206-209**, page **11**. | |
